# Bilateral thalamic and brainstem anaplastic astrocytoma: A case report

**DOI:** 10.1097/MD.0000000000037136

**Published:** 2023-02-02

**Authors:** Hong Zhang, Ping Zhang, Hongbing Nie, Ji Zhang, Jie Li, Xiaoqing Lu, Yaoyao Shen

**Affiliations:** a Department of Neurology, Jiangxi Provincial People’s Hospital, The First Affiliated Hospital of Nanchang Medical College, Nanchang, Jiangxi Province, China.

**Keywords:** anaplastic astrocytoma, bilateral thalamic glioma, brainstem

## Abstract

**Rationale:**

Bilateral thalamic glioma is extremely rare and characterized by strictly limited involvement of bilateral thalami. To investigate its clinical and neuroimaging features, we herein reported a rare case of anaplastic astrocytoma (AA) involving both thalami and the brainstem and reviewed the literature.

**Patient concerns:**

A-33-year-old Chinese woman was referred to our department owing to persistent headache and nausea and vomiting. Neurological examination showed mild cognitive impairment and positive Kernig sign.

**Diagnosis:**

Brain magnetic resonance imaging (MRI) demonstrated asymmetrical and swollen lesions involving both thalami, midbrain and pontine tegmentum, without restricted diffusion or enhancement. On day 7 after admission, she was transferred to the department of neurosurgery and underwent a stereotactic brain biopsy of the right thalamic lesion. Histopathological features and immunohistochemistry were consistent with AA, *IDH* wild-type, World Health Organization grade III.

**Interventions:**

She was administrated with mannitol and glycerin fructose for decreasing intracranial pressure.

**Outcomes:**

In spite of receiving chemotherapy, she died on 2-month after her initial diagnosis.

**Lessons:**

AA involving in both thalami and brainstem is a rare entity with poor prognosis. The clinicians and radiologists should deepen their awareness of the specific MRI feature of bilateral thalamic involvement. When MRI alone is insufficient, the utility of stereotactic biopsy is essential for making a definitive diagnosis.

## 1. Introduction

Gliomas, a heterogeneous group of neoplasms, account for 27% of tumors originating in the central nervous system.^[1]^ Bilateral thalamic glioma (BTG) represents an extremely rare subtype, characterized by strictly limited involvement of bilateral thalami, without any apparent neighboring tumoral tissue.^[2]^ It is estimated that the incidence of BTG is 0.84% to 5.2% among all intracranial tumors.^[3]^ To date, more than 70 cases of BTG have been reported in the literature in the form of case reports and children are the most common.^[4]^ Pediatric patients frequently present with focal neurologic deficits, such as hemiparesis, sensory disturbance, dysmetria, nystagmus, and unsteady gait.^[5]^ Whereas, the prominent clinical presentation in adult patients with BTG is encephalopathy, including dementia, emotional instability, personality changes, apathy, and fatigue.^[6]^ A definitive diagnosis of BTG relies on stereotactic biopsy of brain lesions. Currently, surgery resection of brain tumor is limited due to complex anatomic structures and important functional areas of the bilateral thalami. The most frustrating thing is the therapeutic effect of adjuvant therapies, including radiotherapy and chemotherapy, is not distinct.^[7,8]^ Herein, we describe a rare case of anaplastic astrocytoma (AA) (World Health Organization grade III astrocytoma) involving bilateral thalami and the brainstem who complains of headache, vomiting, and cognitive decline.

## 2. Case presentation

A-33-year-old Chinese woman with a history of hypothyroidism was referred to our department owing to acute onset of headache and loss of appetite for 5 days. The headache was persistent and mainly located in bilateral frontotemporal region, accompanied by dizziness. Her symptoms gradually worsened. Three days later, she complained of nausea and vomiting, without any relief during a break or sleeping. On admission, her vital signs were a temperature of 36.5°C, a heart rate of 65 beats per minute, and blood pressure of 128/88 mmHg. Neurological examination revealed mild cognitive impairment and positive Kernig sign, without any focal neurological deficit. Serological examination yielded hypokalemia (potassium 3.0 mmol/L, reference range 3.5–5.5 mmol/L), hyponatremia (sodium 130 mmol/L, reference range 135–150 mmol/L), and slightly accelerated erythrocyte sedimentation rate (21 mm/h, reference range 0–20 mm/h). Serum concentration of thyroid-stimulating hormone was 12.7 μIU/mL (reference range 0.27–4.2 μIU/mL), triiodothyronine (T3) was 0.79 ng/mL (reference range 0.8–2.0 ng/mL), and free T3 was 3.02 pmol/L (reference range 3.1–6.8 pmol/L). Anti-thyroid peroxidase antibody (TPOAb) and thyroid-stimulating hormone receptor antibody (TRAb) were positive at > 600 IU/mL (reference range < 35 IU/mL) and 1.88 IU/L (reference range < 1.75 IU/L), respectively. Tests of complete blood count, blood glucose, liver and renal function, tumor markers, coagulation function, and d-dimer were all within normal limits.

**Figure 1.**
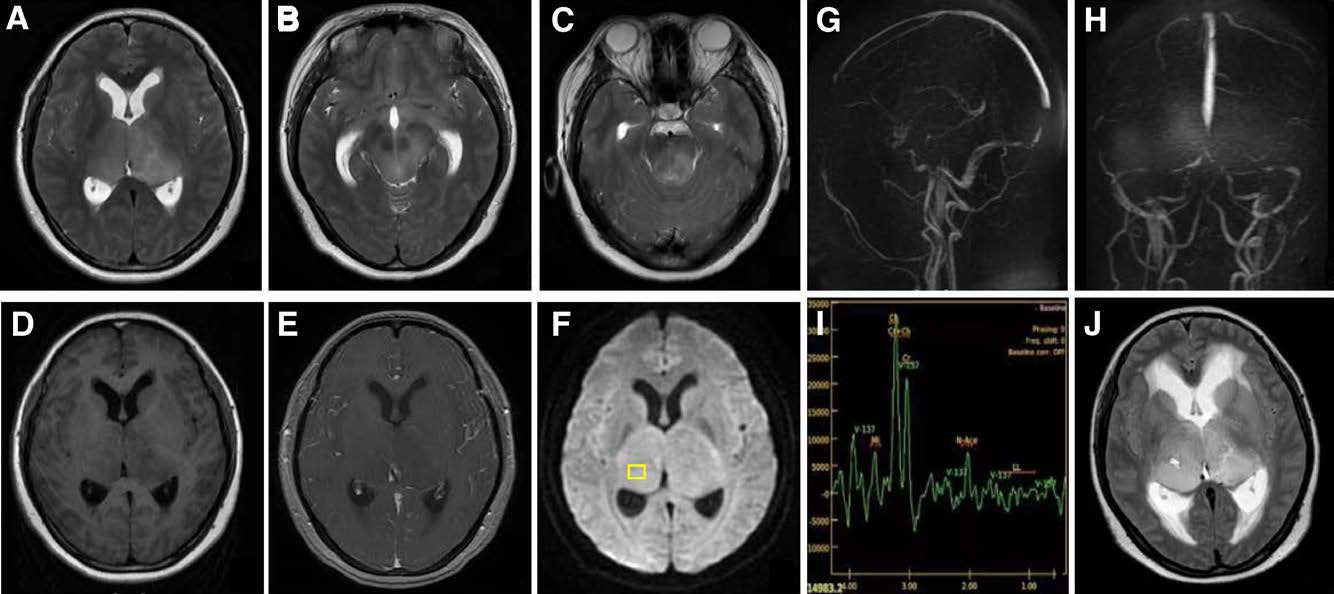
On d 2 after admission, brain MRI demonstrated asymmetrical and swollen lesions involving both thalami, midbrain and pontine tegmentum, hypointense on T1-weighted (D), and hyperintense on T2-weighted images (A–C) without restricted diffusion (F). There was no enhancement within the abnormal area on T1-weighted post-contrast MRI (E). MRV revealed not well visualized inferior sagittal sinus, straight sinus, and bilateral transverse sinuses (G and H). MRS at the level of the yellow square in the lesions revealed higher levels of choline and creatine, and lower level of N-acetyl-aspartate (I). Two mo later, a repeated T2-weighted image demonstrated enlarged lesions and obstructive hydrocephalus (J). MRI = magnetic resonance imaging, MRS = magnetic resonance spectroscopy, MRV = magnetic resonance venography.

**Figure 2.**
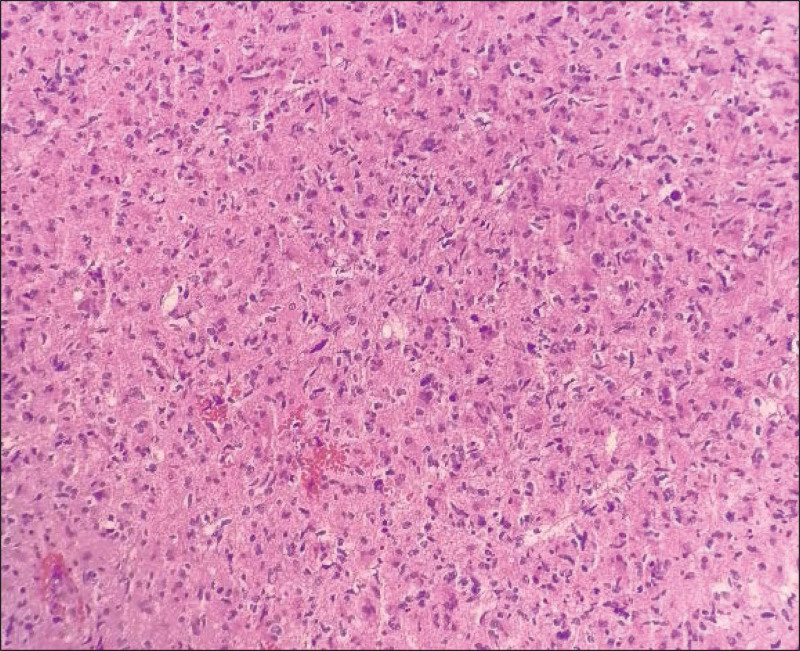
Hematoxylin-eosin-stained sections of paraffin embedded tissue showed pleomorphic astrocytes with hyperchromatic and irregular nuclei without any necrosis or vascular proliferation (original magnification, ×40).

On day 2 after admission, brain magnetic resonance imaging (MRI) demonstrated asymmetrical and swollen lesions involving both thalami, midbrain and pontine tegmentum, hypointense on T1-weighted, and hyperintense on T2-weighted images (Figs. 1A–D) without restricted diffusion (Fig. 1F). There was no enhancement within the abnormal area on T1-weighted post-contrast MRI after gadolinium injection (Fig. 1E). Stenosis or occlusion in the intracranial arteries was not visualized on magnetic resonance angiography. Meanwhile, magnetic resonance venography illustrated not well visualized inferior sagittal sinus, straight sinus, and bilateral transverse sinuses (Fig. 1G and H). Magnetic resonance spectroscopy at the level of the yellow square in the right thalamus revealed higher levels of choline (Cho) and creatine, and lower level of N-acetyl-aspartate (NAA) (Fig. 1I). She was alternately administrated with mannitol and glycerin fructose for decreasing intracranial pressure, so that her headache got partial relief. On day 5 after admission, however, she was somnolence and not oriented to time or place. Her Glasgow Coma Scale was 13/15. In consideration of possible central nervous system tumor, she was transferred to the department of neurosurgery and underwent a stereotactic brain biopsy of the right thalamic lesion on day 7 after admission. Histopathological features (Fig. 2) and immunohistochemistry were consistent with AA, *IDH* wild-type, World Health Organization grade III.

An oncology consultation was subsequently requested. On day 14 after admission, she was transferred to the department of oncology and the followed treatment is only chemotherapy with temozolomide (260 mg/d, day 1–5 each 28-day cycle) and bevacizumab (10 mg/kg every 2 weeks) owing to refusing radiotherapy. She continued to decline and give up the second cycle of chemotherapy. On day 58 after admission, her husband sent her to our hospital again owing to unconsciousness. A repeated T2-weighted image demonstrated distinctly enlarged lesions and obstructive hydrocephalus (Fig. 1J). She was discharged without any further treatment on the same day. Eventually, we received a gloomy message that the patient died on 2-month after her initial diagnosis.

## 3. Discussion

A variety of causes, including neoplasm, metabolic and toxic processes, infection, and vascular occlusion, result in bilateral thalamic lesions. The differential diagnosis can be further narrowed based on previous history, imaging features, and presence of lesions outside the thalami. The artery of Percheron, a rare posterior circulation variant, is a solitary arterial trunk originating from the P1 segment of the posterior cerebral artery and supplying the paramedian thalami and rostral midbrain bilaterally. But our patient has more extensive lesions beyond paramedian area, without hyperintense signal intensity on diffusion-weighted image. Therefore, artery of Percheron infarction could be easily excluded. In our case, brain MRI features are expansion of both thalami, not well visualized venous sinus, and absence of contrast enhancement. Two main causes—deep venous thrombosis and glioma—should be taken into consideration, subsequently. Thrombosis of the deep venous system, such as the internal cerebral veins, vein of Galen, and straight sinus, typically gives rise to a hyperintense T2/FLAIR signal in bilateral thalami and occasionally the basal ganglia. Deep venous thrombosis always demonstrates restricted diffusion without midbrain involvement, which is distinctly inconsistent with this case characteristics. We therefore turn our attention to the diagnosis of glioma. BTG is a rare neoplasm that occurs in both children and adults. Magnetic resonance spectroscopy is of some significance for differential diagnosis. To our knowledge, NAA level is decreased while the neurons are replaced by neoplastic cells. High level of Cho suggests rapid membrane turnover with glial proliferation. The NAA-to-Cho ratio is associated with the degree of tumor infiltration of brain tissue.^[9]^ Hence, the areas of lower NAA/Cho ratio within the tumors are more valuable for precise targeting for the stereotactic biopsy. As described in our case, histopathological results eventually confirmed higher grade of glioma. It worth noting that there is not well visualized intracranial venous sinus, which is largely due to increased intracranial pressure resulting in sinus compression other than thrombosis.

In patients with BTG, most cases are astrocytoma, including diffuse astrocytoma (grade II) and AA (grade III). AA is a diffusely infiltrating, malignant primary brain tumor originating from the neoplastic transformation of astrocytic cells with a median age of onset of 41 years.^[10]^ It has both *IDH* wild-type and *IDH*-mutant variants and the former usually has a more aggressive clinical course with non-optimal response to conventional treatments. *IDH* wild-type glioma frequently exists other molecular alterations, such as epidermal growth factor receptor amplification and telomerase reverse transcriptase promoter mutation, which makes them much more similar to glioblastoma.^[11]^ The BTG patients ranged in age from 3 months to 80 years.^[12,13]^ There is a wide spectrum of clinical presentations in patients with AA including focal neurological deficits, headache, speech disorders, dementia, gait disturbances, seizures, which correspond to the location of the tumor. Generally, the MRI features of AA are T1-weighted hypointense and T2-weighetd hyperintense mass with surrounding vasogenic edema. Nodular enhancement that usually indicates the presence of a high-grade component is frequently observed, although approximately one third of AA display no contrast enhancement.^[14]^ In contrast to oligodendroglioma, AA has homogeneous signal intensity on T2-weighted image and well definable margin, usually without calcifications and cerebral cortex invasion.^[15]^

In our patient, the clinical presentation consists of intracranial hypertension, cognitive impairment, and decreased level of consciousness. In spite of receiving transient relief with dehydration, she eventually died on 2-month after initial diagnosis. Symptoms gradually progress mainly resulting from tumor growth. According to previous researches, AA grows by invasion into normal brain tissue, spreads through the cerebrospinal fluid, and extends beyond a single artery distribution.^[16]^ When thalamus is confronted with glioma, tumor cells originate in the subependymal glia, and hence have primary relation to the medial areas of the thalamus. The tumor can spread across the midline via the interthalamic adhesion and the roof of floor of the third ventricle, and infiltrate into thalamic nucleus, neural tracts, and neighboring tissue. The symptoms always mild in early stages, and gradually aggravate along with tumor growth resulting in intracranial hypertension and focal neurological deficit. In our case, a repeated brain MRI showed severe hydrocephalus, which was due to the direct mass effect resulting compression of the third and fourth ventricles. Progressive dementia was secondary to the involvement of the dorsomedial thalamic nuclei as well as obstructive hydrocephalus. Previous case study demonstrated the mean course of BTG was 2 months and closely related to the degree of malignancy.^[7]^ We want to emphasize that BTG not only involves in both thalami, but also invades into adjacent tissues, such as brainstem as described in our case.

There is no BTG case of radical removal has been reported in the literature because of the complicated thalamic anatomy. However, radiotherapy and chemotherapy just provide short-term advantages in clinical practice, and the roles of those treatments in BTG remain ambiguous. In spite of maximum tumor removal with a combination of adjuvant therapy to prolong patients’ survival, the outcome was still depressing.^[6,8]^ Therefore, neurosurgery operations are limited to stereotactic biopsy and cerebrospinal fluid diversion procedures for cases of associated hydrocephalus. Previously, rare studies have been performed to investigated the prognostic factors for patients with BTG due to low morbidity. Almost all article reported in the form of case report. In the future exploration, multicenter and prospective studies with larger sample sizes on molecular markers are needed to understand this rare disease and its predictive factors.

In conclusion, this case report emphasizes it is important for clinicians and radiologists to recognize the specific MRI feature of bilateral thalamic lesions. BTG is extremely rare entity with generally poor prognosis. It is worth noting BTG is not limited to both thalami, but can also involve in brainstem. Although surgical and adjuvant therapy do not tend to improve the survival of these patients, the utility of stereotactic biopsy is essential for making a definitive diagnosis.

## Author contributions

**Conceptualization:** Hong Zhang, Yaoyao Shen.

**Data curation:** Ping Zhang, Hongbing Nie, Ji Zhang.

**Formal analysis:** Jie Li, Xiaoqing Lu.

**Methodology:** Hong Zhang, Yaoyao Shen.

**Project administration:** Yaoyao Shen.

**Writing – original draft:** Hong Zhang, Yaoyao Shen.

**Writing – review & editing:** Hong Zhang, Ping Zhang, Hongbing Nie, Yaoyao Shen.
